# How perceived Australian sexual norms shape sexual practices of East Asian and sub-Saharan African international students in Sydney

**DOI:** 10.1186/s12889-021-10445-0

**Published:** 2021-02-23

**Authors:** Sylvester R. Okeke

**Affiliations:** Centre for Social Research in Health, UNSW, Sydney, Australia

**Keywords:** HIV/STIs risk, Protective behaviour, Sex talk taboo, Condom, East Asia, Sub-Saharan Africa

## Abstract

**Introduction:**

Sexual norms, real or perceived, shape young people’s sexual behaviour and may constitute one of the greatest challenges in HIV/STIs prevention among this population. This study used sexual script theory to explore how international students in Sydney, Australia – from traditional cultures of East Asian and sub-Saharan African countries – construct home backgrounds and Australian sexual norms and how this may shape their sexual practices during their studies in Australia.

**Methods:**

The study involved face-to-face and telephone semi-structured in-depth interviews. Data were provided by 20 international students who are enrolled in various universities in Sydney. Interviews were audio-recorded, transcribed, coded into NVivo and analysed using reflexive thematic analysis.

**Results:**

Identified patterns in the data showed three themes through which participants perceive sexual norms in Australia as distinctly different from their home country norms. First, participants stated that unlike their home country norms, sexual norms in Australia are permissive. Second, participants hold the view that compared to their home country norms, sex in Australia is largely casual as it is not always attached to love. Some participants revealed that this could shape their own sexual practices during their studies in Australia. Finally, participants noted that compared to their home countries’ norm of sex talk taboo, Australia has an open sexual communication norm; which they believe, enables young people in western societies to easily acquire sexual health information.

**Conclusions:**

Findings provide evidence to support a need for contextualized and effective sexual health services for international students that take account of perceptions around sexual norms and how they can be modified to ensure that sexual practices which these students may engage in, will be managed in a safe and responsible manner.

## Introduction

International students constitute an important population in understanding how socio-cultural norms shape sexual health behaviour in a multicultural setting. Globally, Australia receives the third largest number of international students, with those from African and Asian backgrounds jointly constituting the largest proportion [1]. Australia’s international students’ diversity may also have implications for sexual behaviour and the risk of transmitting HIV and STIs [2, 3]. This makes international students a key population in sexual health concerns in Australia [4–6]. Accordingly, providing responsive preventive, treatment and educational sexual health services to this population, requires a critical understanding of sexual norms that may be shaping their sexual practices. This is important as evidence suggest that international students are not being provided with contextualized sexual health programmes that may address their needs [5, 7, 8] which may also include negotiating conflicting home country and Australian sexual norms.

While there is a lack of official data on the sexual health of international students in Australia, a growing body of evidence shows that this population grapples with sexual health issues and may also be at a higher risk of transmitting and contracting sexually transmissible infections (STIs) and blood-borne viruses (BBVs) than domestic students [2, 6]. Compared to domestic students, international students have lower HIV and STIs knowledge [9, 10] and lower sexual health literacy [11] which have been linked to risky sexual practices and low utilisation of sexual health services [12]. As literature on the sexual health of international students in Australia evolves, a more nuanced understanding of factors shaping their sexual experiences is necessary to guide responsive sexual health programmes.

International students in Australia are a diverse population, as such, socio-cultural and economic variations of home backgrounds could shape their sexual experiences during their studies in Australia. Although previous qualitative studies have explored sex related beliefs, knowledge, attitudes and views of international students in Australia [5, 10, 13], whether and how this shape their sexual practices are yet to be understood. Against this backdrop, beyond attitudes and beliefs, the present study is necessitated by a need for better and more nuanced understanding of how perceived sexual norms in Australia could shape sexual practices and also among a more diverse sample of international students – especially those from African backgrounds – who are underrepresented in existing studies. Consequently, this study recruited participants from East Asian and sub-Saharan African backgrounds across different universities in Sydney.

Although East Asian and sub-Saharan African societies may exhibit a range of diverse sexual norms, these norms are largely conservative and are different from the more liberal sexual norms of Western societies such as Australia [14]. Sexual norms, real or perceived, constitute one of the greatest challenges in HIV and STI prevention among young people [15] as they can promote or prevent risky sexual practices [16]. Thus, one of the most significant goals of youths’ sexual health service would be to understand and modify the perceptions that young people may have about sexual norms. How international students from conservative cultures perceive Australian sexual norms and how this perception may shape their sexual practices during their studies in Australia is poorly understood. To address this research gap, this study uses sexual script theory to explore how international students construct sexual norms around premarital and casual sex, how this may shape their sexual practices while studying in Australia and the implications this may have for HIV/STIs risk and protection.

Sexual script theory – based on the work of Gagnon and Simon (1973) – views human sexual activity as social and learned, rather than as biological and innate. Hence, it situates understanding of sexual behaviour more as socio-cultural than as biological processes [17, 18]. A fundamental proposition of the theory is that sexuality is a learned behaviour that is developed from socio-culturally available messages known as sexual scripts [19]. This theory is used to frame and conceptualise the findings of this study because sexual norms cannot be constructed outside the sexual scripts that participants may have acquired through a process of enculturation and acculturation [18].

Sexual scripts in traditional societies that restrict sex to the confines of marriages, may not prevent young people in these settings from engaging in premarital sex. Thus, paradoxically, premarital sex is common among young people in traditional cultures, notwithstanding that such practice is discouraged by their societal sexual scripts [20]. Consequently, international students from traditional settings may have initiated sex by the time they arrive in Australia. Some may also initiate sex in Australia, as their migration could remove barriers that might have delayed sexual initiation in their home countries. Either way, international students from conservative cultures could change their sexual practices while studying in a more liberal society such as Australia, where premarital and casual sex may be more common [10, 21]. This is because young people from conservative cultures are more susceptible to social influences in their sexual decision-making [22].

Further, according to the Social Norm Theory, individuals have a tendency to behave in ways that aligns with their perceptions of behaviours that are prevalent, accepted or desired in their social environment [22]. Therefore, international students from conservative cultures, may take up sexual practices they may not practice back home. This may also be more likely as there are no longer close parental monitoring and communal living arrangements that may serve to regulate young people’s sexual activities in traditional settings [23].

Moreover, in a technological age, sexual scripts produced by and/or portrayed through social media and new technologies, also influence the construction and reconstruction of sexual norms [24]. For instance, casual sex has been found to be highly scripted and media contents aid this scripting [17, 25]. These scripts portray casual sexual relationship as a normative practice among young people [26] with an Australian government evidence showing that media scripts increasingly sexualize young people [27]. Casual sex among international students raises a concern as young people’s stigmatising attitudes towards condom [28–30], may affect consistent condom use in casual relationships. In the same vein, condom use in casual sexual relationship among international students may also be affected by limited access to sexual health programmes both in their home countries [31, 32] and in Australia [6–8].

Meanwhile, while more liberal cultures such as Australia’s, are characterised by predominantly open sexual discussions, in conservative settings, sexual norms may be marked by sex talk taboo which could further limit opportunities for young people to learn about sex in a safe and regulated environment. This internalised sex talk taboo might affect the extent to which international students from conservative cultures utilise and engage with sexual health services in Australia. Thus, international students from conservative settings could benefit from culturally tailored sexual health services that addresses this barrier. The present study makes a significant contribution by adding to an evolving body of evidence for a need for such target sexual health services.

## Methodology

### Design and paradigm

This qualitative research is informed by the interpretivist paradigm. As such, participants’ perception of sexual norms, both in their home countries and in Australia, are relative and diverse as these perceptions are subjectively constructed through meanings and understandings that participants have developed from individual experiences and social interactions [33]. By building this research on this paradigm, the study sought multiple perspectives on sexual norms among participants. This facilitated a more comprehensive and nuanced understanding of participants experiences [34] and a thick description of the complexity surrounding how their perceived sexual norms may shape their sexual practices while studying in Australia.

Moreover, the researcher – with interest in young people sexual health promotion and HIV/STIs prevention – is a member of the study population and therefore assumes an insider positionality with experiential knowledge of social lives and practices of members of the group. Both the researcher’s interest and positionality are noteworthy because they reflexively and transparently shaped the study’s aims, questions and knowledge claim [35] and are resources rather than biases that affect the findings of the study [36]. This is in line with a core assumption of interpretivist paradigm that posits that researchers’ values shape all aspects of the research process [37]. However, the study findings – which represent a fluid, more informed and nuanced understanding of perceived sexual norms and how they could shape sexual practices of international students – were co-constructed through a dialectical process involving critical dialogue with participants [37].

### Study setting and sample

This qualitative study involved 20 in-depth semi-structured interviews with international students from East Asia and sub-Saharan Africa, conducted between May and August 2019. In order to participate in the study, students needed to be from East Asian or sub-Saharan African country, currently studying on a student visa in undergraduate or postgraduate courses and aged 18 years and above. Additional selection criteria included living in Sydney for at least 3 months prior to the research. The minimum three-month timeframe ensured that the study recruited only participants who are familiar with the Australian social environment.

Participants were recruited using various strategies which involved paper and electronic flyers. Specifically, electronic flyers outlining the study objectives and procedures were shared on different international students’ online platforms. The study also employed snowballing sampling strategy which involved participants sharing the study recruitment material with their networks. Potential participants were asked to contact the researcher via email or by phone to arrange interviews. Participants were recruited from: The University of New South Wales (nine), University of Sydney (four), Macquarie University (three), Western Sydney University (two) and University of Technology Sydney (two). In terms of home backgrounds, participants were recruited from East Asia (China, Indonesia, Japan, Macau, Mongolia, Thailand and Taiwan) and sub-Saharan Africa (Botswana, Cameroon, Kenya, Nigeria, Tanzania, and Zimbabwe). Ethical clearance for the study (HC190215) was granted by a university’s Institutional Review Board – UNSW Human Research Ethics Committee. Informed consent was obtained from all study participants as they provided either written or verbal consent before all the interviews. Written consents were provided by participants who participated in face-to-face interviews while verbal consents were provided by participants who were interviewed over the phone. No personal identifying information of participants was collected. No personal information was also attached to the fieldnote as participants were described by a code involving their background, gender, age and sequence of participation. For instance, for a volunteer from sub-Saharan Africa who identifies as male and is 18 years old, fieldnote and data identification code would be: SSA/M/18/1. This was used to avoid any personal identifying information. Participants that mentioned their country of origin in the course of the interviews had this information deidentified to further ensure strict confidentiality. Participants had their rights, including the right to voluntary participation and to withdraw, fully explained to them. They were also provided with contact details of professional psychologists, should they feel distressed during or after the interview sessions. All the study participants were reimbursed with $40 gift voucher to compensate for the time spent in participating in the research.

### Data collection

The choice of individual in-depth interview as choice of data collection was because interviews may guarantee a more convenient platform for volunteers to engage and discuss sexual issues that are generally treated with secrecy among people from traditional sexual cultures. The interview sessions were guided by a semi-structured interview schedule. The guide consisted of open-ended questions that explored sexual experiences of participants in their home countries and in Australia and factors shaping them. Considering that the sensitive nature of the issues investigated – and among a cultural group where open sexual discussions are discouraged – may have a potential for participants to provide socially desirable views, the researcher consciously built rapport before and throughout the interview process, identified as a member of the group and also shared personal experiences. These encouraged participants to confidently share their views and experiences.

The researcher reflexively ensured that the interview process was dialogic and non-judgmental – both in words and in posture, including tone. Nonetheless, self-distancing mechanism – a strategy that individuals use to psychologically remove themselves from events that happen to them [38] – seems to be evident in some of the interviews as some participants may have used it to mask their involvement in some of the experiences they shared. Besides, volunteers had the choice to be interviewed by someone who share their identifying gender and/or from the same region – East Asia/sub-Saharan but none of the participants utilized these choices. Therefore, the researcher conducted all the interviews in English, and they lasted between 40 and 85 min. The interviews were audio-recorded and transcribed verbatim for the purpose of analysis.

### Data analysis

Based on reflexivity and theoretical sensitivity, data analysis was guided by reflexive thematic analysis (RTA) framework because it allows researcher’s subjectivity, introspection and deeper engagement with the study data in producing knowledge [35, 36]. Also, since data were generated from a culturally and linguistically diverse population, the approach is used because of a need to highlight differences and similarities across the generated data set [36]. Moreover, given that this is an exploratory study, analysis involved inductive, data-driven and bottom-up approach to generate themes from the transcripts [39]. As such, themes in the study are not domain summaries [40] but patterns of shared meaning which are foregrounded around a central meaning-based concept [36]. Data transcripts and field notes were reflexively and reflectively read through multiple times for familiarisation and to gain deeper understanding beyond what they semantically represent. Transcripts were then coded into inductive codes using NVivo. Themes were generated by searching, identifying and refining different codes that collectively represent the codes and provide explanatory framework for the data captured in the codes.

## Results

### Sociodemographic characteristics of participants

The socio-demographic characteristics of the study participants are shown in Table 1. Most participants were aged between 18 and 24 and 25–31 years. Four in five participants were never married while more than one in two (55%) identified as female. Four out of five of the participants reported having had penetrative sex with over three in five participants reporting that they are currently sexually active in Australia.

**Table 1:** Socio-demographic characteristics of participants

	Percentage (%)
**Sex**	
Female	55.0
Male	45.0
**Age**	
18–24 years	40.0
25–31 years	40.0
32 years and above	20.0
**Marital status**	
Single	80.0
Married	20.0
**Background**	
East Asia	50.0
Sub-Saharan Africa	50.0
**Ever had sexual intercourse**	
Yes	80.0
No	20.0
**Sexually active in Australia**	
Yes	65.0
No	35.0

### Identified themes: socio-culturally constructed sexual norms

Data analysis generated three themes representing socio-culturally constructed sexual norms among participants as presented below.

### Permissive sexual norms in Australia

Differences in the construction of sexual norms – in relation to premarital sex – between Australian and home culture norms, was evident in the interviews. Participants believe that compared to their home countries, premarital sex is not only permitted in Australia, but encouraged. They view sexual norms in Australia as permissive, compared to their home countries norms that restrict sex to the confines of marriage or at least a committed relationship that would lead to marriage. This view is shared by participants from both East Asia and sub-Saharan Africa. Notably, most participants preferred this perceived sexual norm that permits premarital sex to the traditional norm of their home countries.

Moreover, the ways in which Australian permissive norms shape sexual practices of participants is complex. Most participants – including those who initiated sex before coming to Australia and those who are yet to initiate sex – admitted that they are likely to be influenced by the more permissive sexual norms of Australia. One of the participants who had initiated sex before coming to Australia, stated:

*I think they [people in Australia] are more sexually active … I would be really sexually active … because people here are very different … I came from a country that’s pretty conservative, whereas, here, it’s a lot more open, which is good* (Female/19/East Asia).

Findings showed that change in sexual practices due to the influence of permissive sexual norms in Australia occurs over time as international students spend more time here.

Based on perspectives from the interviews some international students may be influenced by their perceived Australian norm. One of the participants noted:

*People change the longer they stay here, the longer they are exposed to their culture, the Australian culture. The more they’ve got chances to change their past culture to a brand-new culture that they can have sex easily* (Female/22/East Asia).

Conversely, for some participants, this perceived permissive sexual norms in Australia seems to regulate the extent to which they engage in sexual activities.

*… that’s why even myself, sometimes, I try not to get engaged so much into these things [sexual activities] because their things are quite different from the way we take things. For example, in Africa, you can’t kiss a girl on the road or something like that but here, people kiss each other on the road* (male/27/sub-Saharan Africa)*.*

Furthermore, findings suggest that participants linked perceived view of permissive sexual norms in Australia to casual sex. One of the participants from sub-Saharan Africa noted:

*… like here [Australia], I mean in terms that sex is so casual, … I mean … sex is so casual here [Australia]* (Female/22/sub-Saharan Africa).

The interviews identify the absence of parental monitoring as a catalyst for acculturation into perceived Australian sexual norms. As such, international students tend to engage in behaviours they may not practice back in their home countries. A participant captures this succinctly and presents two anecdotes – about her attendance of night clubs and her friend’s initiation of sex – to explain:

*… a lot of my friends, as soon as they left [home country], they felt like they are free, right, and they become wild and they start to do things that are not okay back home* (Female/19/East Asia).

Continuing, she noted:

*… I feel more open to the cultures here … back in [home country], I wouldn’t even go to [night] clubs because I thought it would be: Oh my God! It’s really a bad thing ... But then now that I’m here almost 12 months, I feel like going to clubs is not really a big thing … I went to clubs four times now* (Female/19/East Asia).

The participant continues, using distancing mechanism, to explain how international students from conservative backgrounds engage in premarital sex because of the more permissive sexual norms of Australia and absence of parental monitoring:

*There’s this one friend, ... She was a very holy person, like oh my God! Like her parents are even more strict than [mine] but then she met this guy and she … had sex with [him]* (Female/19/East Asia).

Notably, from the interviews, it appears that parental monitoring while at home appears to influence how international students from traditional cultures negotiate change in sexual behaviour in their new Australian environment. The interviews data suggests that international students who experienced strict parental monitoring at home tend to feel a great sense of sexual freedom when in Australia. One of the participants stated:

*… if you come from a background where your parents are so strict with you and then you’re like, finally I’m free, then … you would have that [premarital sex]* (female/22/sub-Saharan Africa).

This is also captured in the view of another participant:

*I can actually cite an instance, for example, from the person’s background, there might be pressure from his parents trying to discipline him. So, when he arrives here [Australia], he might say, “ok, what they have been hiding from me, I want to experience it, I want to know what is going on. What it is [all] about”. From there, he might never turn back* (male/19/sub-Saharan Africa).

However, findings also showed that traditional sexual norms prohibiting premarital sex in participants’ home countries appear to be changing due to the influence of Western norms and globalisation. This perception is reproduced in the view of one of the participants:

*People keep, you know, absorbing these Western cultures and, you know, Western … Hollywood movies and stuff. People are becoming more and more [open] nowadays. I believe in Korea and in Japan as well, people care less about [premarital] sex nowadays* (male/22/East Asia).

### Sex not tied to love in Australian sexual norms

There is the view among participants, that compared to sexual norms in their home countries, it is acceptable and normative to separate sex and love in Australia. The interviews showed that while sex and love are portrayed as two sides of the same coin in participants home countries’ norms, they are two different and separate things in Australia and other Western societies. A participant stated:

*I think sex is based on the connection of two people. They love each other … but I think in Australia or other Western world, probably they will separate these two things … no need to base it on the connection to people. Yeah, it’s just pure sex* (female/24/East Asia)

Stressing this ideal condition for sex in home country norms, one of the participants noted:

*I will also say, back home in [home country], I think culturally, you got to seek love before you can engage in sexual activity* (male/32/sub-Saharan Africa).

Participants’ construction of sex as attached to love and affection in their home country norms seems to challenge the conservative sexual norm in which sex is linked to procreation.

Besides, the interviews suggest that participants link pervasive casual sex in Australia to the norm that separates sex from love. For instance, a participant stated:

*… the thing is like it’s also in the culture, the sex involved is actually separate. So, here people have sex with [out] love … like a one-night stand, there are a lot of one-night stands here* (male/22/East Asia).

How this norm potentially shapes sexual practices of participants as regards sexual script theory is complex. In contrast with this theory, some participants maintained that they can never have sex without emotional attachment against the prevailing norm in Australia:

*Because unlike other people out here, I’m very, very cautious and if I’m going to have sex with someone, it’s not going to be a random person. It’s going to be someone that I know for sure this is a person I want to have sex with or this is a person I’m connected with, that I feel vulnerable enough [to], you know, because it’s all about, like sex is a lot about vulnerability. So, if you don’t feel like you could be vulnerable with that person, then that’s not the person* (female/19/sub-Saharan Africa).

On the other hand, some participants revealed that, since having moved to Australia, they now seek non-comital sexual relationships as they perceive to be the norm in Australia.

*I will be blunt and say I’m not rooted to entering into any serious relationship [in Australia], but I will definitely enter into casual dating. I will do casual dating. It will mainly be for sex and not for love* (male/32/sub-Saharan Africa).

### Open sexual discussion in Australia but sex talk taboo in home country

All the participants believed that discussions about sex were held openly in Australia and other western societies, compared to their home country norms which forbid this. Some of the participants described this sex talk taboo norm in their cultures as “awkward”, “absurd” and “weird.” A participant reported:

*I haven’t had to talk to people [here] about sex, but in my opinion, I think they [Australians] may have kind of education, have already told their children about sex and for [home country], we kind of if our children ask about sex, about how baby come to life, … we are kind of afraid about talking sex and even when we talk … we will try to avoid to talk about sex. It’s weird* (female/26/East Asia).

Another participant’s views further reveal that sex talk does not happen in their culture. According to him:

*So, we don’t talk about sex. We don’t talk about sexual behaviour or things in front of the parents. That’s like absurd, it’s like awkward, it’s weird* (male/22/East Asia).

Some of the participants observed that enculturated sex talk taboo makes it almost impossible to discuss sexual concerns with their parents or other adults. Hence, information about issues related to sex is mostly obtained from friends. For instance, a participant revealed:

*All my sexual information has come from my best friend … It has come from my best friend, because my best friend has had a boyfriend since she is 12 years old, so I have heard a lot of things from her* (female/22/East Asia).

Learning about sex or seeking sexual health information from friends or peers – who might not be knowledgeable or from unsafe pornographic materials – rather than in a safe sexual healthy environment might lead to risky sexual health behaviour. For instance, one of the participants revealed:

*… I learn from her because she has shared with me her relationship and sexual things with her boyfriend … so I know quite a lot and I need to share that the first time I watched a pornography that my best friend shared with me and she said, her boyfriend gets her to watch some of the videos … so it was my first time to watch pornography … and I just felt so embarrassed to watch it at that time. I think I was 15 years old at that time* (female/22/East Asia).

A majority of the participants believe that sex talk taboo contributes to sexual health problems like unplanned pregnancy, HIV and STIs risks. One of the participants observed:

*May be the culture of not being very open … in Africa, they don’t talk at all about sex openly … so we just do like hiding, which in a way … can be risky … I guess the culture of not being open … may be like [it’s] not helping* (male/27/sub-Saharan Africa).

This view is also reiterated by another participant:

*I think it is very important to teach kids what is involved in sex, not only for the enjoyment part as well as like a baby-making activity, but there can be risk for getting or transmitting STIs or unwanted pregnancy, so yeah, hiding and stigmatizing sex-related talks or topics is one of the keys or the missing link of the things that should be improved or changed* (male/39/East Asia).

A similar view is shared by a younger participant from a different East Asian country:

*I actually like the western culture attitude towards sex. I think being open is really good, because you know how like in [home country] people don't talk about sex and as a result they don't teach about sex in schools and stuff and as a result we have a very high rate of teenage pregnancy and it’s because people aren’t informed. These teenagers they are not knowledgeable and when you don't get taught in class, you try it out yourself in the real world (female/19/East Asia).*

## Discussions of findings

In line with the sexual script theory, most of the study participants expressed the belief that they might be influenced by Australian sexual norms to adopt sexual practices that their new Australian environment permits. Moreover, the study results also reflect the paradoxical sexual culture of conservative settings, where premarital sex is common; notwithstanding that socio-cultural norms prohibit such practice [20]. Therefore, since most of the participants were already sexually active in their conservative settings, where premarital sex is discouraged, now that they are in a more liberal Australian society, they may continue this practice and may even become more sexually active with the potential of engaging in risk-related behaviours. For instance, the likelihood of engaging in casual sex (which may also be unprotected), once in Australia may be reinforced by participants’ normative view of such practice in Australia.

Therefore, casual sex may be one of the notable perceived Australian sexual scripts that may have serious implications for HIV and STI transmission. Though the risk of HIV transmission may be low in Australia, STI prevalence is relatively high and also on the increase [41, 42] and infection with an STI can increase the risk of HIV [43]. According to the study findings, participants believe that premarital sex in Australia is mostly casual, compared to their home countries; where it is supposed to occur in a romantic relationship that may lead to marriage. This normative view of casual sex in Australia might result in casual sexual activity among international students, which when unprotected, increases the risk of HIV and STI transmission [44]. While protected casual sexual relationship may not be a risk for STI/HIV transmission [45, 46], consistent condom use has been found to be low among migrant groups and this could be the case among international students from countries with conservative sexual norms which stigmatise condom [30]. Specifically, multiple partner relationships occurring along with no or low condom use have been reported among young East Asian immigrants in Canada [47] and sub-Saharan African immigrants in Australia [48].

Notably, some participants hold the view that it is difficult, if not impossible for international students to maintain a conservative sexual lifestyle in Australia. This confirms the finding in a recent study among international students in an Australian university [5]. Therefore, perceived Australian sexual scripts could be used by these students to rationalise any risky sexual lifestyle they may adopt while studying in Australia. This is especially the case considering that compared to their home countries, in Australia, they no longer experience any parental monitoring or communal lifestyle which often acts to regulate young people’s sexual activities in traditional settings [23]. Participants also reported that international students who experience harsh parenting back home may be more likely to engage in regular and potentially risky sexual practices while studying in Australia. This is consistent with findings of previous research that have also investigated the effect of parenting style on sexual behaviour of young people [49, 50].

Additionally, an interesting dimension of the study findings may be an emergent sexual script in contemporary traditional societies that justifies premarital sex when it is attached to love. Participants disclosed that premarital sex in their home countries occurs within the context of love, especially when there is a prospect of marriage. This, they believe, is not the case in Australia and other western societies where people have sex, even if they are not emotionally attached to each other. Participants’ reconstruction of premarital sex as an expression of love may signal a new sexual script in contemporary East Asian and sub-Saharan African societies that is different to the traditional sexual script which ties sex to procreation with little or no regard to emotional attachment [51, 52]. By constructing sexual norms that portray sex as an expression of love, young people in contemporary traditional settings might be seeking to achieve a modern sexual identity that is distinct from the traditional past [53]. This is achieved through the process of deconstruction and reconstruction which young people may use to challenge and seek to displace hegemonic traditional sexual norms. The study findings support earlier research conducted among young people in an East-Asian setting which also showed that these participants perceived premarital sex as proof of emotional attachment in romantic relationships [54].

Also, the study found that traditional cultural norms of sex talk taboo and sexual secrecy may compound HIV/STI risks among international students from conservative settings. Limiting choices of young people in seeking sexual clarifications due to sex talk taboo might have a negative impact on HIV and STIs protective behaviour. Participants expressed the belief that compared to their home countries, Australia has open sexual communication norms. They indicated a preference for the Australian norms which, they believe, help young people to acquire sexual health information; whereas in conservative societies, sexual discussions are shrouded in secrecy and even seen as a taboo [55]. However, while some participants indicated that perceived Australian permissive sexual cultures can influence them to become more sexually active in Australia, it is unclear whether they will also adapt to Australian open sexual discussion norm especially in seeking sexual health services. Therefore, sex talk taboo may act as a barrier to students accessing sexual health information for risk prevention as well as appropriate treatment for sexual health concerns during their studies in Australia.

Sex talk taboo in conservative settings is usually connected with the fear that openly discussing sexual issues with young people, may arouse their interest in sex [55]. Thus, this secrecy could foreclose open discussion of sexual issues and concerns in a safe environment. The study data suggest that sex talk taboo may impair or totally discourage sex education at home and in schools. It might also result in young people seeking sexual health information from peers who might equally not possess accurate sexual health knowledge. This could increase the risk of HIV and STI transmission [56] as well as unplanned pregnancies and complications resulting from clandestine abortions [57, 58]. However, seeking sexual health information from friends or peers, as found in this study and in line with previous evidence in Australia [59], provides evidence of the usefulness of peer education for sexual health services for international students as it would allow international students to maintain their confidentiality. Meanwhile, it is important for further studies to explore, more closely, how sex talk taboo may impact sexual health information and care seeking among international students from conservative cultures. Similarly, further research is needed to understand how race, gender, cultural norms and sexual identity intersect to influence sexual practices among this population.

### Study limitations

The results of this study must be understood within some caveats. First, the study found a high level of consensus in the responses of participants; with little nuances in the construction of sexual norms among participants between the two regions (between group) and across countries that make up each region (within group). This consensus may be attributed to the fact that both regions are largely conservative, and as such, may share similar norms. Moreover, participants volunteered and so are unlikely to be representative of broader international students’ population.

## Conclusion

Participants reported that the norms of their home countries differ to those in Australia where sex is perceived as more casual and permissive as well as more openly discussed. Findings from this study suggest that the way international students perceive sexual norms in Australia might shape their sexual practices. Therefore, there is a need for providing contextualised and culturally sensitive sexual health services for this population that will enable them to manage any new sexual practices they may adopt while studying in Australia in a safe way.

## Data Availability

The datasets used and/or analysed during the current study are available on reasonable request.
